# PEG-Dependent Tunable Degradation and Curcumin Release from Curcumin-Based Biomedical Polyurethanes

**DOI:** 10.3390/biom16050640

**Published:** 2026-04-24

**Authors:** Man Wang, Hongying Liu, Wei Zhao, Huafen Wang, Yuwei Zhuang, Ran Zhang, Zhaohui Liu, Nengwen Ke, Sichong Chen

**Affiliations:** 1 High & New Technology Research Center of Henan Academy of Sciences, No. 56 Hongzhuan Road, Zhengzhou 450002, China; 2 West China School of Nursing, Sichuan University, No. 37, Guoxue Alley, Chengdu 610041, China; 3 The Collaborative Innovation Center for Eco-Friendly and Fire-Safety Polymeric Materials (MoE), National Engineering Laboratory of Eco-Friendly Polymeric Materials (Sichuan), College of Chemistry, State Key Laboratory of Polymer Materials Engineering, Sichuan University, Chengdu 610064, China

**Keywords:** polyurethane, PEG-dependent, curcumin delivery, biodegradability, biocompatibility

## Abstract

Curcumin, a plant-derived polyphenolic compound, exhibits diverse pharmacological activities such as antioxidant, anti-inflammatory, anticancer, neuroprotective, and cardiovascular protective effects, and is widely used in food, medicine, and other fields. However, its poor water solubility and easy oxidative degradation limit its extensive application in biomedicine. To solve these problems, a series of biomedical polyurethanes (Cur-PU) with similar molecular weights but different PEG contents were successfully synthesized using HO-PCL-OH and HO-PEG-OH as soft segments and curcumin as a chain extender. The results indicated that increasing the PEG content reduced the T^1^_m_, T^1^_c_, and H^1^_c_ of Cur-PU, along with a slower crystallization rate and lower crystallinity. More importantly, a higher PEG content decreased the water contact angle but increased water solubility and water uptake, which, combined with reduced crystallinity, enhanced hydrophilicity, swelling ratio, curcumin release rate, and degradation rate in an enzymatic solution and pH 8.0 buffer. Thus, precise regulation of Cur-PU’s degradation and curcumin release was achieved by controlling the PEG content. Biocompatibility tests confirmed that Cur-PU exhibited excellent antioxidant and antibacterial activities, making it a highly promising biomedical material.

## 1. Introduction

Curcumin (1,7-bis-(4-hydroxy-3-methoxyphenyl)-hepta-1,6-diene-3,5-dione) is the primary bioactive component extracted from the rhizomes of *Curcuma* genus plants (Zingiberaceae family), such as *Curcuma longa*, *Curcuma phaeocaulis*, and *Curcuma aromatica*. Classified as a diketone polyphenolic compound, it displays a diverse range of significant pharmacological activities, including antioxidation [1], anti-inflammation [2], anticancer effects [3], neuroprotection [4], and cardio protection. Furthermore, curcumin has been recognized as a Generally Recognized as Safe (GRAS) substance by the U.S. Food and Drug Administration (FDA), thereby conferring substantial application prospects in the food and pharmaceutical industries. However, the practical application potential of curcumin is severely constrained by its intrinsic limitations. Firstly, it exhibits extremely poor water solubility (solubility < 1 μg/mL in water), which makes it difficult to dissolve in the acidic environment of the gastrointestinal tract, consequently resulting in dissolution-rate-limited absorption. Secondly, curcumin undergoes rapid metabolism in the liver and intestinal wall, where it is mainly converted into inactive metabolites through glucuronidation and sulfation pathways. Thirdly, its short in vivo half-life and rapid systemic clearance lead to remarkably low oral bioavailability, such that effective concentrations are hardly detectable in plasma even at conventional dosages.

To address this issue, Jiaxing Huang [5], Thelma Akanchise [6], Qianhua Feng [7], Wenmei Zhao [8], Fenting Lei [9], et al. have utilized the unique cell membrane–mimetic bilayer structure of liposomes to fabricate a series of lipid-based nanocarriers, including liposomes, solid lipid nanoparticles (SLNs), and nanostructured lipid carriers (NLCs), via the encapsulation of curcumin. These lipid-based nanocarriers not only enhance the water solubility and stability of curcumin but also facilitate its fusion with cell membranes, promoting the intracellular delivery of curcumin and thereby significantly increasing the distribution concentration of curcumin in tissues such as the liver and spleen. Notably, these carriers exhibit excellent biocompatibility, high physical stability, and a relatively simple preparation process. They can enable efficient control over the release rate of curcumin and prolong its in vivo circulation time. Nevertheless, they suffer from certain inherent limitations: relatively low drug loading capacity, potential mild gastrointestinal irritation induced by some lipid materials, and the risk of crystalline transformation during long-term storage, which may compromise the long-term efficacy of curcumin release.

In contrast, the drug loading capacity of curcumin nanoparticles using polymers as carriers has been significantly enhanced. This improvement is primarily attributed to the presence of polar groups (e.g., ester bonds, amino groups, and hydroxyl groups) on the polymer molecular chains. These polar groups can form multiple intermolecular interactions with curcumin, such as hydrogen bonding, hydrophobic interactions, and π-π stacking, which enable the “anchoring” of curcumin molecules inside or on the surface of the polymer matrix rather than simple physical encapsulation. This anchoring effect substantially reduces the leakage and loss of curcumin during the preparation process, thereby improving the actual drug loading capacity. Furthermore, polymer carriers can self-assemble or be fabricated into nanoparticles/nanomicelles with core–shell, porous, or cross-linked network structures via methods such as emulsion-solvent evaporation. These polymer-based nanostructures possess abundant internal cavities or hydrophobic microdomains that can accommodate a large number of curcumin molecules. Notably, the pore size of the cross-linked network can be regulated by adjusting the cross-linking degree, which not only allows for the effective encapsulation of curcumin but also achieves stable drug loading through the swelling–shrinking behavior of the polymer matrix. For instance, the curcumin solid dispersion prepared with PVP as the carrier exhibited a 590% increase in bioavailability in rats compared to conventional tablets. Cihui Tian [10], Wuxiu Cao [11], Jingjing Fu [12], and Dong-Wei Wang [13], et al. conjugated curcumin with hyaluronic acid, sodium alginate, and chitosan to fabricate specifically recognizable nanoparticles. These nanoparticles not only achieve tumor-targeted delivery but can also be degraded by hyaluronidase, with no toxic or side effects from the degradation products. Gang Wang [14], Maryam Ghaffari [15], and Yan Gong [16], et al. loaded curcumin onto hydrogel nanoparticles based on polylipoic acid, polydopamine, polyamide dendrimers, and polylysine. These hydrogel nanoparticles not only improved the bioavailability of curcumin but also enabled the gradual release of encapsulated nanoparticles as the hydrogel degraded, significantly prolonging the release duration of curcumin and exhibiting superior antioxidant and anti-inflammatory effects. This polymer-based carrier strategy offers advantages such as high structural stability, excellent drug loading capacity, and the feasibility of precise targeting through polymer molecular modification. However, their widespread application is constrained by several inherent limitations: some synthetic polymers exhibit poor biodegradability, which may lead to in vivo accumulation and potential toxicity over the long term; additionally, the immunogenicity of polymer materials may trigger immune responses in the organism. Consequently, the development of polymer carriers with excellent biocompatibility, favorable biodegradability, and high drug loading capacity is urgently required.

In response to the aforementioned requirements, degradable polyester-based polymers have attracted increasing attention. These polymers exhibit excellent biocompatibility and can degrade into small-molecule organic acids (e.g., lactic acid, glycolic acid) via ester bond cleavage. As FDA-approved injectable carrier materials, they are highly suitable for the long-term sustained release and targeted delivery of curcumin. Studies have demonstrated that PLGA carriers can stably encapsulate curcumin through hydrophobic interactions, effectively isolating curcumin from the destructive gastrointestinal environment and significantly enhancing its in vitro stability and in vivo circulation time. Compared with PLGA, PLA possesses higher crystallinity and a relatively slower degradation rate, making it more suitable for curcumin delivery scenarios requiring long-term sustained release. However, PLA exhibits strong hydrophobicity, and pure PLA carriers have limited encapsulation efficiency and drug loading capacity for curcumin. Thus, surface modification or a composite with other materials is often required to optimize their performance. In addition to PLGA and PLA, PCL, polyhydroxybutyrate (PHB), and other degradable polyesters are also commonly used in curcumin carrier research. For instance, Yalin Zhang et al. [17] fabricated a five-layer biomimetic membrane with gradient pore sizes using PCL as the raw material via electrospinning technology. After loading curcumin, this membrane achieved “multi-functional performance of a single membrane”: it not only could manage wound exudate via unidirectional capillary force-driven transport to avoid reflux but also enabled the sustained release of curcumin at the wound site, exerting antioxidant, anti-inflammatory, and pro-angiogenic effects. Animal experiments showed that it shortened the wound healing time by nearly 5 days and reduced scar formation by more than 50%. Additionally, MaLing Gou et al. [18] encapsulated curcumin into methoxy poly(ethylene glycol)-b-poly(ε-caprolactone) (MPEG-PCL) micelles via a one-step nanoprecipitation method. The resulting micelles exhibited an encapsulation efficiency of 99.16 ± 1.02% and a drug loading capacity of 12.95 ± 0.15% and could inhibit colon cancer growth by suppressing angiogenesis and directly killing cancer cells.

Although the aforementioned approaches have successfully combined biodegradable polyesters with curcumin, achieving optimal curcumin release efficacy in the short term, the binding force between the two is insufficient when applied to long-term drug delivery due to the surface modification strategy. This inadequacy inevitably impairs the durability of sustained curcumin release. To ensure the long-term efficacy of curcumin in drug delivery, chemical bulk modification has emerged as a promising strategy. However, research in this field remains relatively scarce, with limited literature available for reference. Previously, our research group synthesized curcumin-modified polyurethane materials via a one-pot two-step method, using HO-PCL-OH as the soft segment and curcumin as the hard segment [19]. Nevertheless, it was found that due to the slow degradation rate of PCL, curcumin embedded in the main chain was difficult to release, failing to exert the desired antibacterial, antioxidant, and anti-inflammatory biological activities. In the present work, we adopt HO-PCL-OH and HO-PEG-OH as dual soft segments, with curcumin serving as a chain extender, to synthesize a curcumin-modified polyurethane material with a tunable degradation rate and excellent antibacterial performance via bulk modification. Notably, this strategy enables the tailored design of the polyurethane’s hydrophilic–hydrophobic properties, mechanical performance, and degradation rate by regulating the mass ratio of the dual soft segments, thereby meeting practical application requirements. Furthermore, the antibacterial and antioxidant activities of polyurethane can be precisely regulated by controlling the reaction ratio of curcumin. This ensures that the material can stably penetrate the gastrointestinal tract and release curcumin in the acidic or alkaline gastrointestinal environment, ultimately exerting the desired biological effects.

## 2. Materials and Methods

### 2.1. Materials

The ε-caprolactone (ε-CL) monomer (purity ≥ 99%) was supplied by J&K Chemical (Beijing, China). Curcumin (analytical reagent, AR, ≥98%), Hexamethylene diisocyanate (HDI ≥ 99%), N,N-Dimethylformamide (DMF ≥ 99.8%, Water ≤ 50 ppm (by Karl Fischer titration, MkSeal 500 mL), Sodium hydroxide (NaOH, AR), Phosphoric anhydride (P_2_O_5_, >98.5%), 1,4-Butanediol (BDO ≥ 99%), Anhydrous sodium phosphate dibasic (Na_2_HPO_4_ ≥ 99%), and Sodium Phosphate Monobasic (NaH_2_PO_4_ ≥ 99.9%) were purchased from Macklin (Shanghai, China). Bisdemethoxycurcumin (≥98%, RG), Polyethylene glycol (HO-PEG-OH, average M_n_3350), Stannous octoate (Sn(Oct)_2_, purity 92.5–100%), and hexafluoroisopropanol (HFIP, AR) were obtained from Adamas (Shanghai, China), Aladdin chemical (Shanghai, China) and Tansoole (Shanghai, China), respectively. Methanol (MeOH, AR) and chloroform (CHCl_3_, AR) were procured by Kelong Reagent Corp. (Chengdu, China) and used without further purification. Prior to use, ε-CL monomer and BDO were purified via vacuum distillation over calcium hydride (CaH_2_). Curcumin and bisdemethoxycurcumin were dried in an oven at 40 °C to a constant weight before use.

### 2.2. Methods

Nuclear Magnetic Resonance (NMR) spectra were recorded in *d*6-DMSO using a 400 MHz Agilent Technologies spectrometer operated at room temperature (Santa Clara, CA, USA). Chemical shifts (δ) for ^1^H were referenced to the tetramethylsilane (TMS) internal standard (0.00 ppm) and the *d*_6_-DMSO solvent peak (2.50 ppm), respectively. Fourier transform infrared (FTIR) spectra were collected on a Nicolet 6700 spectrometer (Thermo Fisher Scientific, Agawam, MA, USA) in the wavenumber range of 600~4000 cm^−1^. Gel permeation chromatography (GPC) was performed on an HLC-8320 system (Tosoh Corporation, Tokyo, Japan) equipped with a refractive index detector. DMF was used as the eluent at a flow rate of 1 mL min^−1^ and a column temperature of 30 °C. Thermogravimetric analysis was conducted on a TG STA 2500 Regulus thermogravimetric analyzer (NETZSCH, Selb, Germany) under a nitrogen flow of 50 mL min^−1^. Samples were heated from 40 to 700 °C at a heating rate of 10 °C min^−1^. Differential scanning calorimetry (DSC) measurements were carried out using a TA DSC25 instrument (TA Instruments, Milford, MA, USA) in an aluminum pan under a N_2_ flow of 50 mL min^−1^. The thermal protocol was as follows: the samples were first heated to 80 °C at 5 °C min^−1^ and held for 2 min to eliminate thermal history, then cooled to −80 °C at a 5 °C min^−1^, and finally reheated to 80 °C at the same rate. The crystallinity index (χ) of Cur-PU was calculated using the following equation:

(1) $$\chi_{c,PCL}=\frac{{\Delta H}_{m,PCL}}{{\Delta H}_{0,PCL}\;\times \varphi }\times 100\%\;\;\;\;\;\chi_{c,PEG}=\frac{{\Delta H}_{m,PEG}}{{\Delta H}_{0,PEG}\;\times \varphi }\times 100\%$$

where ΔH_m,PCL_ is the experimental melting enthalpy, and φ is the weight fraction of the corresponding component in the blend. ΔH_0,PCL_ = 139 J/g for PCL was used according to the reported enthalpy of melting of 100% crystalline PCL [20], and ΔH_0,PEG_ = 163.18 J/g for PEG was used according to the reported enthalpy of melting of 100% crystalline PEG (3350).

Mechanical properties of samples were evaluated using a Gotech Testing Machine (Dong Guan) (Gotech AI-7000-M, Guangzhou, China) at a crosshead speed of 10 mm min^−1^ at room temperature. X-ray diffraction (XRD) patterns were acquired on a Bruker AXS D8 Advance diffractometer (Bruker, Karlsruhe, Germany) using Cu Kα radiation (λ = 0.15418 nm) at 40 kV and 5 mA. Scans were performed in the 2θ range of 5–50° with a step size of 0.02° and a scanning rate of 5° min^−1^. The water contact angle of the samples prepared by hot-pressing was measured using a contact angle goniometer (Krüss DSA100, Hamburg, Germany). Scanning electron microscopy (SEGMA, Baden-Württemberg, Germany) was used to observe the surface morphologies of specimens. UV/vis absorption spectra were recorded on a Shimadzu UV-3600 Plus spectrophotometer (Shimadzu, Tokyo, Japan) using 1 cm pathlength quartz cuvettes. Fluorescence spectra were obtained using the F-7000 Fluorescence spectrophotometer (Hitachi, Beijing, China) at room temperature. The slit width was 5 nm and 2.5 nm for excitation and emission.

### 2.3. The Shape–Memory Experiment

The shape–memory experiment of Cur-PU films was conducted using the three-point bending method. Firstly, rectangular Cur-PU splines were placed in an oven at 60 °C for 5 min to maintain a constant temperature. The splines were then folded, and the temperature was rapidly reduced to 25 °C under a certain pressure to fix the temporary shape (θ_0_). Finally, the splines with the temporary shape were replaced in a constant-temperature water bath at 60 °C for recovery, and the recovered shape (θ) was obtained. The shape-fixity ratio (R_f_) and shape-recovery ratio (R_r_) in the shape–memory test are calculated using Formula (2), where θ and θ_0_ represent the angle size of the spline after fixation and recovery, respectively. Each sample was measured three times, and the average value was taken.

(2) $$R_{f}=\frac{180-\theta_{0}}{180}\times 100\%,\;R_{r}=\frac{\theta -\theta_{0}}{180}\times 100\%$$

### 2.4. Moisture Content, Water Solubility, and Swelling Behaviors

The moisture content (MC), water solubility (WS), and swelling behavior (SR) of the Cur-PU film were determined by referring to Qin’s method with some modifications [21,22]. The films (2 cm × 2 cm) were placed in a constant temperature and humidity oven (25 °C, 50% RH) for 1 d and their initial mass was recorded as W_0_. The samples were then placed in an oven at 85 °C to dry to a constant weight, and the weight after drying (W_1_) was recorded. The MC of the film was then calculated according to Equation (3). Subsequently, the film was immersed in distilled water at room temperature for 24 h before being dried to constant weight at 85 °C and weighed as W_2_. The WS of the film was calculated using Equation (4). Another film sample was taken to record the initial mass (W_0_), then placed in distilled water at room temperature for 24 h. After that, excess water was sucked from the swollen film using a filter paper and weighed as W_3_. The SR of the sample was calculated using Equation (5) [23,24].

(3) $$MC(\%)=\frac{W_{0}-W_{1}}{W_{0}}\times 100\%;$$

(4) $$WS(\%)=\frac{W_{1}-W_{2}}{W_{1}}\times 100\%;$$

(5) $$SR(\%)=\frac{W_{3}-W_{0}}{W_{0}}\times 100\%;$$

### 2.5. In Vitro Drug Release Behavior of Cur-PU Sample

#### 2.5.1. Standard Curve of Cur

An appropriate amount of Cur was weighed and dissolved in absolute ethanol to prepare a Cur solution with a concentration of 1.0 mg/mL. The Cur solution was diluted with PBS (pH 7.4, 1.0 wt% SDS) to a concentration of 1–100 μg/mL, and the UV absorption values at 427 nm were measured.

#### 2.5.2. In Vitro Drug Release

An appropriate amount of Cur-PU was placed in a 50 mL centrifuge tube, and 15 mL of PBS (pH 7.4, containing 1% SDS, *w*/*v*) was added. The sample was incubated in a shaker at 37 °C with a shaking speed of 50 rpm. At predetermined time intervals, 2 mL of the supernatant was collected, and an equal volume of fresh release medium was replenished. The UV absorption spectra of the supernatant from three parallel samples were measured using a UV–Vis spectrophotometer. The concentration of curcumin in the release medium was calculated based on the standard calibration curve of curcumin. The cumulative release percentage of curcumin was calculated using the following equation:

Cumulative release percentage (%) = M_t_/M_0_ × 100% (6)

where M_t_ is the total amount of released curcumin at a certain time point, and M_0_ is the initial total amount of curcumin loaded in the hydrogel [25].

### 2.6. pH Sensitivity of Cur Solution and Cur-PU Films

The pH sensitivity of Cur solution and Cur-PU films was determined according to the method of Liu with some modifications [26]. An appropriate amount of Cur was dissolved in ethanol and added separately to buffer solutions of different pH (2–12). The UV spectra of the Cur solutions were analyzed using a UV–Vis spectrophotometer in the wavelength range of 400–700 nm. The Cur-PU film samples were cut into small pieces of about 1.5 cm and immersed in buffer solutions of different pH (2–12), removed after ten minutes, and the surface water was dried with filter paper. Optical pictures of the solutions and films were recorded using a camera [27].

### 2.7. Degradation Experiment

Degradation tests were conducted using a 100 mL silk reagent bottle in a controlled environment chamber set at 25 °C and 60% relative humidity. For the air, UPW, artificial pancreatic juice [28], and pH 6.0/8.0 phosphate buffer solutions medium, the solution was replaced every week. At the end of each predetermined incubation period, the supernatant was decanted, and the remaining specimen was thoroughly washed with deionized water, dried under vacuum at room temperature for three days to a constant weight, and then weighed to record the mass loss before and after degradation, calculated using Formula (7). (W_0_: the mass of the sample before degradation; W_i_: the mass of the sample after degradation.)

(7) $$Mass\;loss\left(\%\right)=\frac{W_{i}-W_{0}}{W_{0}}\times 100\%$$

### 2.8. Antioxidant Activity

The antioxidant activities of the Cur-PU sample in vitro were measured with a DPPH assay [29]. DPPH was dissolved in absolute ethanol at a concentration of 0.05 mg mL^−1^. The Cur-PU sample (100 mg) was soaked in 1 mL of UPW at 37 °C, and the supernatant was taken as the Cur-PU extract. A total of 60 μL of Cur-PU extract and 940 μL of absolute ethanol were added to the DPPH solution (2 mL). After mixing for 30 min in the dark, the UV absorbances of DPPH solutions at 517 nm were detected to calculate the clearing efficiency of DPPH. Three parallel samples were used in each test.

### 2.9. Cytotoxicity Test

Cur-PU samples were immersed in DMEM supplemented with 10% PBS and incubated for 24 h under 5% CO_2_ at 37 °C. C2C12 cells were seeded into 96-multiwell plates at a density of 5 × 10^4^ cells per well in 500 μL of 10% PBS DMEM and cultured for 24 h at 37 °C. The old culture medium was then aspirated, and 200 µL of extract from each specimen group was added. The plates were further incubated for 24 h, 48 h, and 72 h under the same conditions, after which cell viability was evaluated using a CCK-8 assay [30].

### 2.10. Hemolysis Assay

Rat blood (1 mL) was centrifuged at 3000 rpm for 10 min, and the upper serum layer was discarded. The red blood cell pellet was washed three times with PBS. Subsequently, 20 µL of RBCs was mixed with 500 µL of PBS extract containing different Cur-PU samples. Ionized water and PBS were used as positive and negative controls, respectively. The mixtures were incubated at 37 °C for 4 h, then centrifuged at 3000 rpm for 10 min. The absorbance of hemoglobin in the supernatant was measured at 540 nm using a UV–Vis spectrophotometer, and the hemolysis rate (HR) was calculated using Formula (8) [31,32], where A_t_, A_pn_, and A_nn_ are the absorbances of the test sample, positive control, and negative control, respectively.

(8) $$HR\left(\%\right)=\frac{\left(A_{t}-A_{nc}\right)}{\left(A_{pc}-A_{nc}\right)}\times 100\%$$

### 2.11. In Vitro Antibacterial Test

#### 2.11.1. Preparation of Bacterial Suspension and Samples

In a sterile laminar flow hood, *Staphylococcus aureus* and *Escherichia coli* were separately streaked onto Luria–Bertani (LB) agar plates using pipette tips. The plates were then incubated at 37 °C for bacterial rejuvenation. A single colony was picked and inoculated into 10 mL of liquid LB medium, followed by shaking incubation at 37 °C for 12 h until the late exponential phase. The bacterial culture was centrifuged at 8000 rpm for 5 min at 4 °C, washed twice with PBS, and resuspended in an equal volume of PBS to obtain a bacterial suspension with a concentration of 10^8^ CFU/mL.

#### 2.11.2. Zone of Inhibition

The zone of inhibition of hydrogels was evaluated according to the reference. At first, 20 μL of bacteria solution (10^8^ CFU/mL) was added to petri dishes (60 mm in diameter) containing Luria–Bertani agar gels, and then gently smoothed using a coating rod to cover the full surface. Then, a circular piece (6 mm in diameter) of the agar gel was removed by incision to reveal the underlying polystyrene. Cur-PU film was then prepared in the cavity of agar plates following the method mentioned before. Then, the plates were incubated at 37 °C for 24 h and 96 h and then observed and photographed. Each sample was repeated three times [33].

#### 2.11.3. Determination of Minimum Inhibitory Concentration (MIC)

A total amount of 10 μL of the bacterial suspension was pipetted into different samples and incubated at 37 °C for 2 h. Subsequently, 100 μL of the mixture was transferred onto sterile LB solid agar plates, evenly spread over the surface, and incubated in a 37 °C incubator for 24 h. The bacterial growth status was then observed and recorded. MIC was determined using the two-fold serial dilution method. The samples were serially diluted two-fold with sterile liquid LB medium to yield a final concentration range of 1.95–500 μg/mL. The bacterial suspension, cultured to the mid-logarithmic phase, was adjusted to a concentration of 1 × 10^6^ CFU/mL, and 10 μL of this suspension was thoroughly mixed with the samples at each concentration. A total amount of 200 μL of the resulting mixture was inoculated into a 96-well plate and incubated at 37 °C for 48 h. The absorbance values were measured at a wavelength of 600 nm using a microplate reader at 3 h intervals [34,35].

## 3. Results

### 3.1. Chemical Structure Characterization of Cur-PU

**Figure 1:**
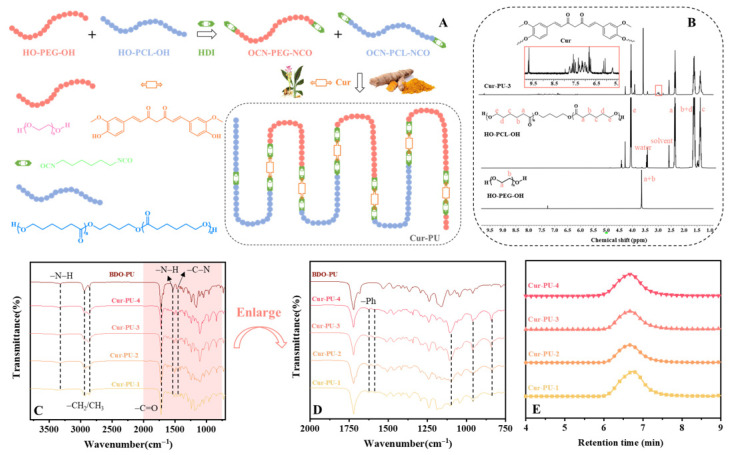
(**A**) The synthetic procedure of Cur-PU; (**B**) the ^1^H NMR spectra of Cur-PU-3. The FTIR spectra (**C**,**D**) and GPC traces (**E**) of Cur-PU and BDO-PU samples.

Figure 1A outlines the synthetic protocol for Cur-PU polyurethane materials. Herein, HO-PCL-OH and HO-PEG-OH served as soft segments, and curcumin was introduced as a chain extender to synthesize a library of Cur-PU polyurethanes with comparable molecular weights yet variable PCL segment contents. For comparative analysis, BDO-PU polyurethane was prepared using BDO as the chain extender to delineate the specific impact of curcumin incorporation on polyurethane physicochemical properties. NMR spectroscopy was employed to characterize HO-PCL-OH, HO-PEG-OH, and Cur-PU (Figure 1B); accordingly, the characteristic resonances of HO-PEG-OH and HO-PCL-OH were assigned to the chemical shifts at 3.65 ppm and 3.98 ppm, respectively. The integral area ratios of these diagnostic peaks were subsequently utilized to quantify the relative contents of PEG and PCL segments in Cur-PU. On this basis, the molar fraction, mass fraction, and crystallinity of PCL segments within Cur-PU were computed, with the resultant data systematically compiled in Table S1. Figure 1C,D depicts the FTIR spectrum of Cur-PU. It can be observed that the absorption peak of –N=C=O around 2250 cm^−1^ disappears, indicating the complete reaction of isocyanate groups [36]. The absorption peak at 3319 cm^−1^ in Cur-PU corresponds to the stretching vibration of the N–H bond, while the absorption peaks at 1626 cm^−1^ and around 1515 cm^−1^ correspond to the stretching vibrations of the aromatic C=O and C–C bonds in curcumin (Cur), respectively. In addition, the peak appearing at 817 cm^−1^ for Cur-PU is attributed to the stretching vibration of –C–C– on the curcumin backbone. Figure 1E shows that the M_n_ of Cur-PU ranges from 38 to 42 kDa, which is higher than that of its precursors, with relatively narrow polydispersity indexes (Ð ≤ 1.72, Table S1). Collectively, the NMR, FTIR, and GPC results of Cur-PU confirm the successful grafting of Cur components into the Cur-PU polyurethane materials.

### 3.2. Thermal, Behavioral, and Crystallinity Properties of the Cur-PU Films

**Figure 2:**
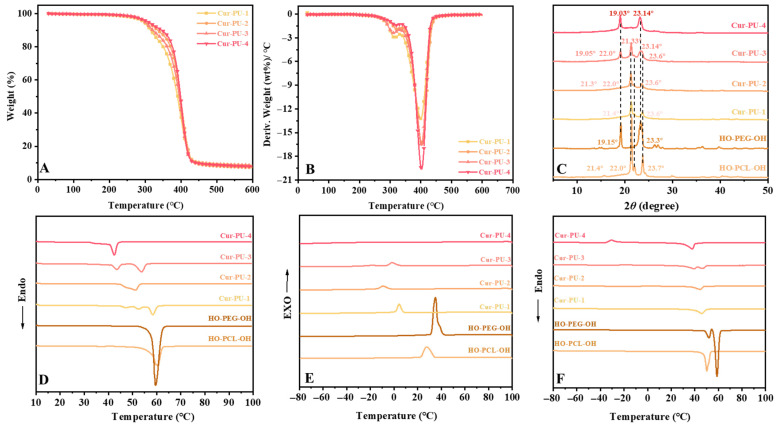
The representative TG (**A**) and DTG (**B**) curves; (**C**) the X-ray diffraction spectra. DSC thermograms: the first heating scans (**D**), the first cooling scans (**E**), and the second heating scans (**F**) of Cur-PU, HO-PEG-OH, and HO-PCL-OH prepolymer.

The thermal stability of Cur-PU, BDO-PU, and prepolymers HO-PEG-OH and HO-PCL-OH was characterized by DSC and TG (Figure 2, Tables S1 and S2). The T_5%_ and T_max_ values of all Cur-PU samples were higher than those of BDO-PU (288.1 °C and 356.51 °C, respectively). The improved thermal stability arises from the introduction of curcumin as a hard segment, which facilitates the formation of a denser hydrogen bonding network. During degradation, aromatic moieties in curcumin stabilize the polymer backbone via conjugation and form a barrier char layer, providing a dual stabilization effect. Consequently, Cur-PU shows two distinct decomposition stages in the DTG curves (Figure 2B).

A comparative analysis of the DSC curves (Figure 2E,F) of HO-PCL-OH and HO-PEG-OH prepolymers reveals that the T_g_ of Cur-PU falls between that of HO-PCL-OH (−60.58 °C) and HO-PEG-OH (−43.62 °C). Meanwhile, the T^1^_m_, T^2^_m_, and H^1^_m_, H^2^_m_ of Cur-PU are significantly lower than those of the two prepolymers. With increasing the PEG content, T^1^_m_, T^1^_c_, and H^1^_c_ of Cur-PU decrease gradually, and no crystallization peak is observed for Cur-PU-4. This phenomenon can be attributed to two factors: the bulky steric structure of curcumin restricts the crystallization of PCL and PEG segments, and the structural randomness of polyurethane increases as the contents of PCL and PEG become comparable. As shown in Figure 2C and Figure S3, when PCL segments are dominant, the XRD patterns of Cur-PU-1 and Cur-PU-2 match well with HO-PCL-OH, indicating that crystallization mainly occurs in the PCL domains. With increasing the PEG content, the crystallinity (χ_c_) of Cur-PU-2 (38.98%) is slightly lower than that of Cur-PU-1 (46.67%), as randomly distributed PEG segments disrupt the ordered packing of PCL segments and hinder crystallization. A similar trend is seen in Cur-PU-4: although its XRD pattern is consistent with HO-PEG-OH, its crystallinity (48.39%) is lower than that of Cur-PU-3 (59.14%). In contrast, Cur-PU-3 shows two sets of diffraction peaks corresponding to both PCL and PEG segments due to their co-crystallization, thus exhibiting the highest crystallinity among the Cur-PU series.

### 3.3. Mechanical Properties of the Cur-PU Films

**Figure 3:**
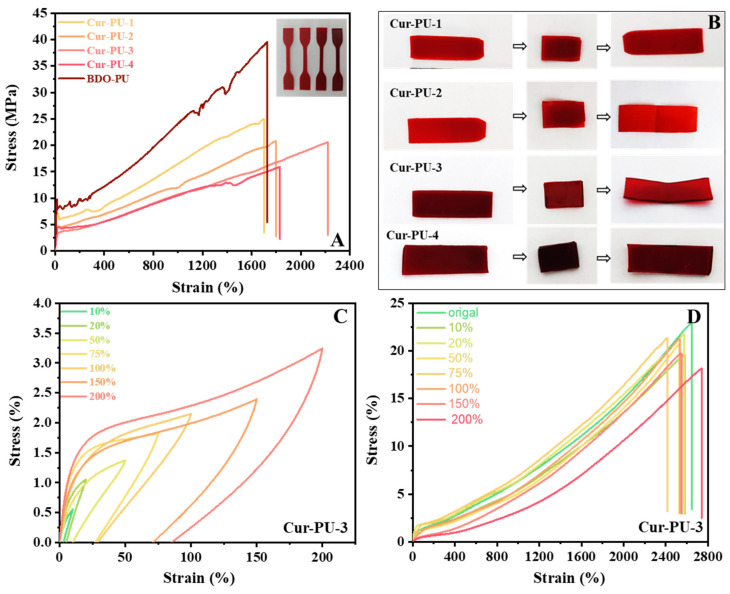
The stress–strain curves (**A**) and shape memory (**B**) of Cur-PU film. (**C**) The cyclic stress–strain curves of Cur-PU-3 at a strain of 10%, 20%, 50%, 75%, 100%, 150% and 200% for five continuous cycles. (**D**) The stress–strain curves of Cur-PU-3 after being cycled and relaxed for 24 h.

As shown in Figure 3A, curcumin incorporation imparts a reddish-brown color to Cur-PU films, which deepens gradually with increasing PEG content. Despite significantly higher crystallinity than BDO-PU, the crystallization rate of Cur-PU is hindered by HO-PEG-OH (Cur-PU-1 and Cur-PU-2) or HO-PCL-OH (Cur-PU-4) segments, as well as curcumin’s bulky steric structure, resulting in lower tensile strength than BDO-PU. Additionally, Cur-PU’s tensile strength and hardness decrease continuously with rising PEG content, and notably, Cur-PU-3’s tensile curve shows no yield point, indicating typical elastomeric behavior.

Cyclic tensile tests under small deformations (Figure 3C and Figure S1, Table S4) reveal obvious first-cycle hysteresis loss in Cur-PU-3, arising from microstructural damage, hydrogen-bond dissociation, and other stretching-induced effects—characteristics of the Mullins effect. In the subsequent four cycles, the loading curves nearly overlap with the prior unloading curves, and the hysteresis loss gradually declines, demonstrating excellent elastic recovery (up to 80%). Its tensile strength decreases slightly with increasing cycles, primarily due to the disruption of partial physical cross-links during continuous stretching and delayed recovery, leading to gradual performance degradation. However, after 24 h of storage at room temperature, re-tensile tests (Figure 3D, Table S5) show that Cur-PU-3 regains over 90% of its original tensile strength and elongation at break, except for the 200% strain. This is because hydrogen-bonded physical cross-links can gradually reconstruct under small deformations, while those damaged by large-strain cyclic loading are difficult to fully restore, leading to relatively inferior mechanical properties. Three-point bending results (Figure 3B, Table S6) further confirm that all Cur-PU films exhibit shape fixation and recovery ratios above 90%, indicating excellent shape–memory performance.

### 3.4. The Cur Release of Cur-PU Films

**Figure 4:**
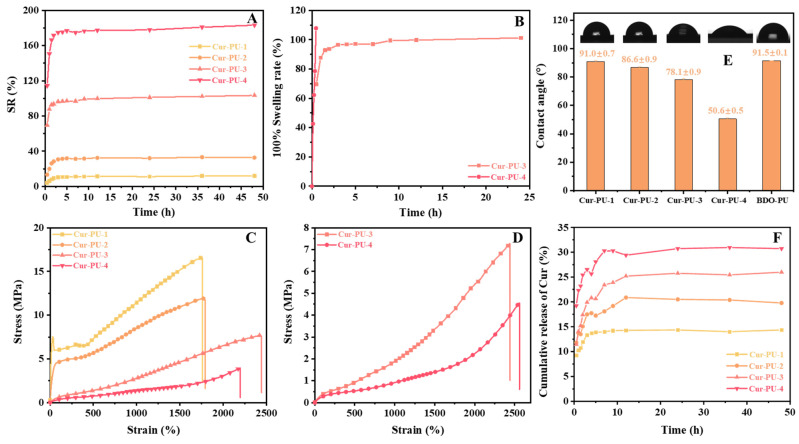
(**A**) The equilibrium swelling plots of Cur-PU in deionized water at 37 °C; (**B**) 100% swelling plots of Cur-PU-3 and Cur-PU-4 in deionized water at 37 °C. (**C**) The stress–strain curves of Cur-PU after equilibrium swelling. (**D**) The stress–strain curves of Cur-PU-3 and Cur-PU-4 after 100% swelling. (**E**) The water contact angle of Cur-PU. (**F**) The release curve of Cur from Cur-PU.

The water contact angle, water content, and swelling results are presented in Figure 4A,E and Figure S2, and Table S7. With increasing the PEG content, the water contact angle of the polyurethanes decreases from 91.0° to 50.6°, transitioning from hydrophobic to hydrophilic. Accordingly, Cur-PU-4 exhibits the highest water content and water solubility (10.1% and 9.89%, respectively). After 48 h of water immersion, the swelling ratios of Cur-PU increase gradually with higher PEG content. Cur-PU-4 shows the highest swelling degree and the shortest time to reach 100% swelling (Figure 4B and Table S8). Upon further immersion, the maximum swelling ratios of Cur-PU-1 and Cur-PU-2 reach 11.84% and 32.63%, respectively, while those of Cur-PU-3 and Cur-PU-4 reach 103% and 183%. The mechanical properties of the samples at 100% swelling ratio were tested (Figure 4D and Table S9). Cur-PU-3 and Cur-PU-4 display significantly lower tensile strength than the original samples, with no obvious yielding, demonstrating typical elastomeric characteristics. Although the increased thickness after water immersion reduces tensile strength, the films maintain excellent toughness and elasticity, consistent with the results for the equilibrium swelling samples (Figure 4C).

To investigate the Cur release profile from Cur-PU, samples were immersed in PBS (pH 7.4, 1% SDS, *w*/*v*) at 37 °C with shaking at 50 rpm. Supernatants were collected at predetermined intervals, and Cur release rates were calculated using the Cur standard curve (Figure S3), with the results shown in Figure 4F and Table S10. Cur is released rapidly within the first 10 h and nearly reaches equilibrium after 48 h, with a maximum release rate of 30.72%. The Cur release from Cur-PU films does not reach 100%, which can be attributed to two main factors. First, curcumin is incorporated into the polyurethane backbone as a chain extender via covalent bonding rather than simple physical doping or encapsulation. Second, the Cur release proceeds in two stages: the first stage is mainly driven by the swelling of Cur-PU. In the initial period, the Cur-PU film absorbs a large amount of water, leading to the rapid release of a considerable portion of curcumin. The second stage mainly depends on the degradation of the Cur-PU film. With the degradation of PCL and PEG segments, curcumin moieties on the polyurethane backbone are gradually released. Since the degradation rate of the PCL segments is relatively slow, the release in this stage proceeds gradually.

### 3.5. pH Responsiveness of the Cur-PU

**Figure 5:**
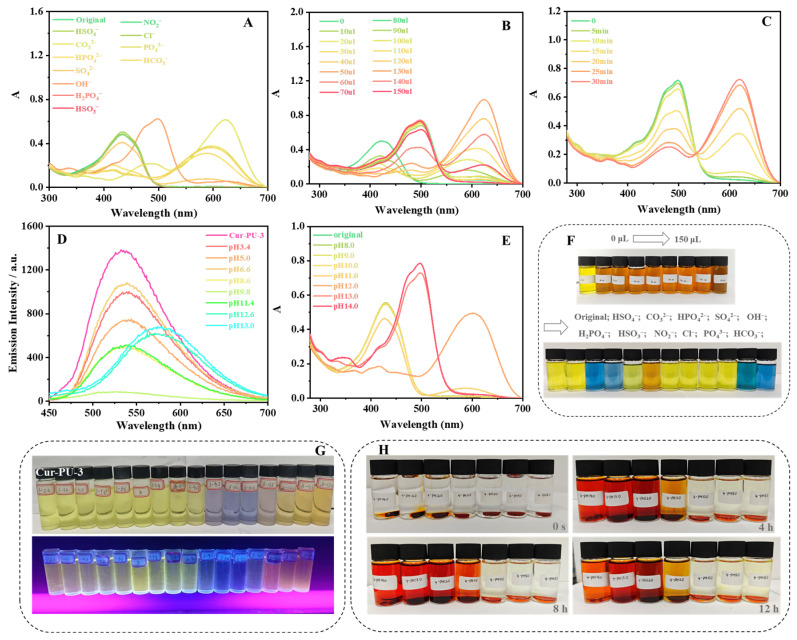
(**A**) The absorption spectra of Cur-PU-3 solution (0.016 mg/mL in DMSO) upon the addition of 150 μL of different anions; (**B**) Cur-PU-3 solution with different volumes of OH^−^; (**C**) Cur-PU-3 solution with 150 μL OH^−^ for different settling times at room temperature. (**D**) The emission spectra of Cur-PU-3 solution (0.016 mg/mL in DMSO) with different pH levels. (**E**) The absorption spectra of Cur-PU-3 solution upon the addition of different pH levels. (**F**) Images captured of the Cur-PU solution with 150 μL of different anions and the Cur-PU-3 solution with different volumes of OH^−^. (**G**) Images captured of the Cur-PU-3 solution upon the addition of different pH levels and within different pH solutions. (**H**) The color change in Cur-PU-3 film after being placed in different pH solutions for various durations.

Upon dropwise addition of various anion solutions into the Cur-PU solution, only OH^−^ induced a distinct color transition from yellowish-green to orange (Figure 5F), with a significant redshift of the absorption maximum from 422.0 nm to 498.5 nm (Figure 5A and Table S11). This is mainly ascribed to enol–keto tautomerism of curcumin moieties in Cur-PU-3. Under strong alkalinity, enolic hydroxyl groups and vinylic protons in curcumin units interact with OH^−^ and dissociate to form oxygen anions. The increased lone-pair electron density on these anions enhances the auxochromic effect, markedly increasing both the maximum absorption wavelength and absorbance of curcumin in alkaline media.

To further explore the spectral and color changes with OH^−^ addition, 10~150 μL of OH^−^ was gradually added to the Cur-PU-3 solution. The absorption peak at 422.0 nm gradually decreased, and a new band appeared at 623.0 nm. This 623.0 nm peak weakened and vanished with increasing OH^−^ concentration, while a new peak at 498.0 nm emerged and intensified (Figure 5B and Table S12), corresponding to the solution color changing from yellowish-green to orange (Figure 5F). At low OH^−^ concentrations, curcumin’s enolic hydroxyl groups are preferentially deprotonated. With increasing OH^−^, active protons dissociate sequentially, continuously increasing the maximum absorption wavelength and absorbance. Sufficient OH^−^ leads to the full deprotonation of all enolic hydroxyl and vinylic protons, with the solution’s absorption wavelength and intensity reaching their maximum values.

When Cur-PU-3 was exposed to solutions of different pH values, similar absorption changes were observed (Figure 5E and Table S14). Beyond the UV–Vis colorimetric response, Cur-PU-3’s fluorescence color showed a ratiometric transition from pale yellow to pink (Figure 5G), with an obvious redshift in the emission spectrum (Figure 5D and Figure S4 and Table S15). However, the OH^−^-treated Cur-PU-3 solution was unstable, fading gradually with corresponding spectral changes within 30 min (Figure 5C and Table S13) due to the reversible enol–keto tautomerism of curcumin.

After immersing Cur-PU disks in solutions of different pH for 4 h, the medium changed from colorless to reddish orange. The color intensified with increasing alkalinity, accompanied by gradual thinning of the Cur-PU disks (Figure 5H). This is mainly attributed to the gradual cleavage of ester and urethane linkages in Cur-PU, which promotes the sustained release of curcumin moieties. After 12 h of immersion in pH 13 or pH 14 media, the disks directly fractured into fragments or degraded into thin films that could not be recovered for characterization. These results indicate two key features: first, Cur-PU films are degradable, with a degradation rate strongly dependent on environmental pH, where higher alkalinity accelerates degradation. Second, curcumin moieties are progressively released as ester and urethane bonds degrade, enabling favorable biological functions. The biological performance is closely related to pH: stronger alkalinity and longer immersion times lead to higher curcumin release and thus better biological efficacy.

### 3.6. In Vitro Degradation Behavior of Cur-PU Films

**Figure 6:**
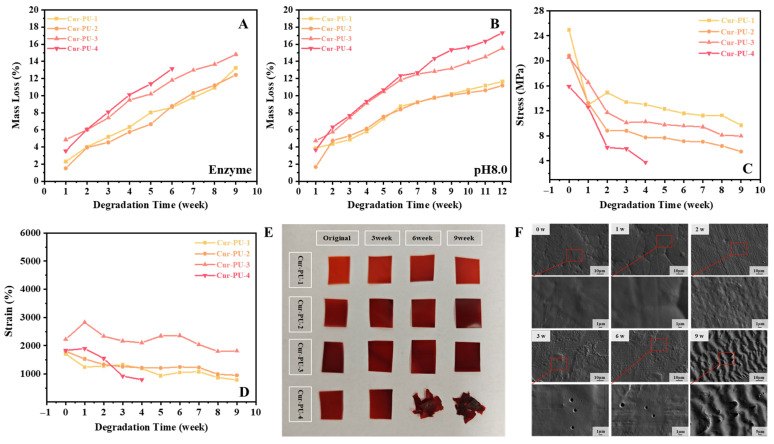
The weight loss of Cur-PU after degradation in enzyme (**A**) and pH 8.0 phosphate buffer solution (**B**) for 9 weeks. The change in stress (**C**) and strain (**D**) of Cur-PU after degradation in enzyme for 9 weeks. (**E**) Images captured of the Cur-PU after degradation in enzyme for 9 weeks. (**F**) The SEM photographs of Cur-PU-3 after degradation in enzyme for 9 weeks.

The mass loss of Cur-PU films in air, deionized water, bioenzyme solution, and pH 8.0 phosphate buffer is presented in Figure 6A,B and Figure S5. The mass loss of Cur-PU increases with the PEG content due to enhanced hydrophilicity, with Cur-PU-4 showing the highest mass loss. Mass loss is most significant in the pH 8.0 buffer, reaching 18% for Cur-PU-4 after 12 weeks, as the alkaline environment reacts with curcumin’s enol structure to accelerate polyurethane degradation. After 4 weeks in bioenzyme solution, the Cur-PU-4 dumbbell specimens cracked and could not be subjected to mechanical testing. After 6 weeks, the square samples degraded into small irregular fragments (Figure 6E), some of which were lost during washing or too tiny for weighing. After 9 weeks, the remaining samples showed significantly reduced strength and elongation at break (Figure 6C,D, Table S16). Strength dropped sharply in the first 3 weeks and then slowed; elongation followed a similar trend, with all samples except Cur-PU-4 retaining over 700% elongation. This is attributed to proteases and lipases accelerating Cur-PU degradation, resulting in increased mass loss, especially for Cur-PU-4. SEM images reveal that bioenzymes etch pores of various sizes on the film surface (Figure 6F), causing an initial sharp drop in mechanical properties. Since degradation proceeds via surface-controlled layer-by-layer erosion without damaging the bulk interior, Cur-PU still maintains high toughness after 9 weeks.

### 3.7. The Bioactivity of Cur-PU Films

**Figure 7:**
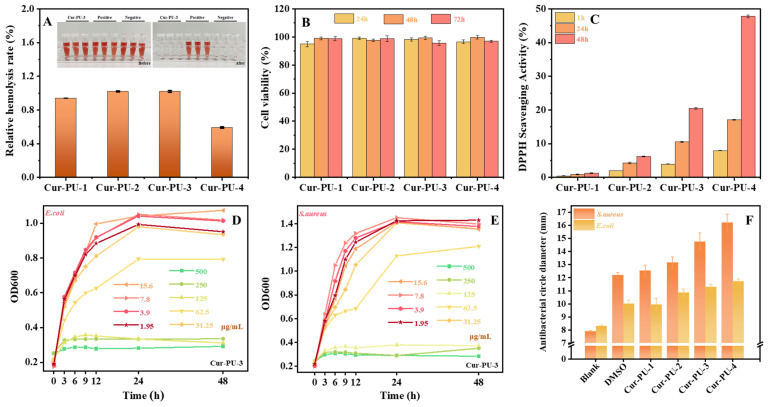
The hemolysis test (**A**), cell viability of C2C12 cells (**B**), and ROS-scavenging efficiencies (**C**) of the extracts of Cur-PU film. The MIC of *E. coli* (**D**) and *S. aurens* (**E**) of Cur-PU-3. (**F**) The diameter of the antibacterial zone of the extracts of Cur-PU film.

Figure 7A and Figure S6 and Table S18 present the hemolysis ratios of red blood cells incubated with Cur-PU extracts. No obvious hemolysis was observed in any group, and all relative hemolysis ratios were below 1.2%, far below the 5% safety threshold. These results confirm that Cur-PU films possess excellent hemocompatibility. Cell proliferation assays using C2C12 cells showed that cell viability remained above 95% after 24 h, 48 h, and 72 h of incubation with Cur-PU extracts (Figure 7B and Table S18). Moreover, cell numbers increased noticeably with prolonged incubation time. Cytotoxicity tests of Cur-PU films also revealed cell viability above 95%, indicating favorable cytocompatibility for both the films and their degradation products. Notably, Cur-PU films exhibited outstanding antibacterial activity. The minimum inhibitory concentrations (MIC) of the extracts against *S. aureus* and *E. coli* were both 125 μg/mL(Figure 6D,E and Figure S7 and Table S19). Inhibition zone tests further demonstrated significantly stronger antibacterial efficacy against *S. aureus* than against *E. coli* (Figure 6F). In addition, antibacterial performance improved with increasing the PEG content in the Cur-PU, as higher PEG content enhances hydrophilicity and thus promotes faster curcumin release within the same extraction period. Antioxidant assays supported this mechanism: the DPPH radical scavenging capacity of each sample increased with time and also rose with higher PEG content at the same time point (Figure 7C and Table S17). Overall, these results demonstrate that the integration of curcumin moieties effectively endows polyurethanes with excellent antibacterial and antioxidant properties. By adjusting the PEG segment content, the curcumin release rate can be finely regulated, allowing Cur-PU to exert ideal biological functions and improve curcumin bioavailability.

## 4. Conclusions

A series of curcumin-based biomedical polyurethane materials (Cur-PU) was synthesized by employing HO-PCL-OH and HO-PEG-OH as soft segments, with curcumin serving as a novel chain extender. The successful incorporation of curcumin and the quantitative regulation of PEG content were systematically verified via ^1^H NMR, FTIR, and GPC analyses. The effects of PEG content on the physicochemical properties, degradation, and drug release behavior of Cur-PU were extensively investigated. The TG results demonstrated that all Cur-PU materials exhibited excellent thermal stability, with T_5%_ exceeding 250 °C. DSC measurements revealed that increasing the PEG content suppressed the crystallinity of the polyurethanes, which significantly enhanced hydrophilicity, thereby accelerating the swelling ratio, curcumin release, and degradation rates. Notably, the Cur-PU-4 sample showed rapid degradation and favorable in vitro excretion potential. Furthermore, all Cur-PU materials displayed superior biocompatibility (cell viability > 90%, hemolysis rate < 1.2%) as well as potent antioxidant and antibacterial activities. This study indicates that Cur-PU materials possess tunable physicochemical properties and promising potential for biomedical applications.

## Data Availability

The data presented in this study are available on request from the corresponding author.

## Supplementary Materials

The following supporting information can be downloaded at https://www.mdpi.com/article/10.3390/biom16050640/s1. Figure S1. The cyclic tensile properties of Cur-PU-3 film under different deformations. Figure S2. The moisture content (MC) and water solubility (WS) of the Cur-PU film. Figure S3. The UV Standard Curve of Curcumin. Figure S4. The emission spectra of Cur-PU-1, Cur-PU-2, and Cur-PU-4 solution (0.016mg/mL in DMSO) with in different pH. Figure S5. The changes of mass of Cur-PU samples at different degradation time in air and upw. Figure S6. The hemolysis test of Cur-PU-1, Cur-PU-2, and Cur-PU-4. Figure S7. The MIC of *E.coli* and *S.aurens* of Cur-PU-1, Cur-PU-2, and Cur-PU-4 film. Table S1. The chemical composition, molecular characteristics and TG results of Cur-PU and BDO-PU sample. Table S2. The DSC results of Cur-PU; BDO-PU, HO-PCL-OH and HO-PEG-OH prepolymer. Table S3. The XRD results of Cur-PU; HO-PCL-OH and HO-PEG-OH prepolymer. Table S4. Mechanical performances, water contact angle of Cur-PU and BDO-PU samples. Table S5. The of cyclic tensile properties of Cur-PU-3 films under different deformations and mechanical properties of the films after relaxed 24h at room temperature. Table S6. Detailed data of the ratio (R_f_) and recovery ratio (R_r_) of Cur-PU and BDO-PU samples. Table S7. The moisture content (MC) and water solubility (WS) of the Cur-PU film. Table S8. The swelling behaviors (SR) of the Cur-PU film. Table S9. Mechanical performances of Cur-PU samples after swelling behaviors. Table S10. The cumulative release of Cur of Cur-PU samples. Table S11. The UV absorption data of Cur-PU-3 solution with 150 μL of different anions. (Cur-PU-3: 0.016 mg/mL; solvent: DMSO; anions: 0.1 mol/L). Table S12. The UV absorption data of Cur-PU-3 solution with different volumes of OH^−^. (Cur-PU-3: 0.016 mg/mL; solvent: DMSO; OH^−^: 0.1 mol/L). Table S13. The UV absorption data of Cur-PU-3 solution with 150 μL OH^−^ for different settling times at room temperature (Cur-PU-3: 0.016 mg/mL; solvent: DMSO; OH^−^: 0.1 mol/L). Table S14. The UV absorption data of Cur-PU-3 solution with upon the addition of different pH. (Cur-PU-3: 0.016 mg/mL; solvent: DMSO; pH: 0.1 mol/L). Figure S15. The emission data of Cur-PU solution with in different pH. (Cur-PU-1: 0.016 mg/mL; solvent: DMSO; pH: 0.1 mol/L). Table S16. The changes of Mechanical performances of Cur-PU samples at different degradation time in artificial pancreatic juice. Table S17. The antioxidant activity of Cur-PU film. Table S18. The cell viability of C2C12 cells cultured for 24 h, 48 h, and 72 h in extracts of the of Cur-PU and BDO-PU film and the hemolysis test of Cur-PU and BDO-PU film. Table S19. The antibacterial test of Cur-PU film.
