# Supplementary material for: PEG-Dependent Tunable Degradation and Curcumin Release from Curcumin-Based Biomedical Polyurethanes
Source: Biomolecules. 2026 Apr 24;16(5):640. doi: 10.3390/biom16050640 (PMC13204639; doi:10.3390/biom16050640)
Supplement: Supplementary file 1 [file biomolecules-16-00640-s001.zip › biomolecules-4264674-supplementary.pdf]

Supporting Information

# PEG-Dependent Tunable Degradation and Curcumin Release from Curcumin-Based Biomedical Polyurethanes

Man Wang[†1]; Hongying Liu[†2];Wei Zhao[1]; Huafen Wang[1]; Yuwei Zhuang[1]; Ran Zhang[1]; Zhaohui Liu[1]; Nengwen Ke[2*]; Sichong Chen[3*];

[1] High & New Technology Research Center of Henan Academy of Sciences, No. 56 Hongzhuan Road, Zhengzhou 450002, P.R. China;

[2] West China School of Nursing, Sichuan University, Chengdu 610041, China;

[3] The Collaborative Innovation Center for Eco-Friendly and Fire-Safety Polymeric Materials (MoE), National Engineering Laboratory of Eco-Friendly Polymeric Materials (Sichuan), College of Chemistry, State Key Laboratory of Polymer Materials Engineering, Sichuan University, Chengdu 610064, China; chen-sichong@scu.edu.cn (S.-C.C);

* Correspondence: chensichong@scu.edu.cn (S.-C.C); kenengwen@scu.edu.cn (N.-W. Ke);

Figure S1. The of cyclic tensile properties of Cur-PU-3 film under different deformations.

Figure S2. The moisture content (MC) and water solubility (WS) of the Cur-PU film.

Figure S3. The UV Standard Curve of Curcumin.

Figure S4. The emission spectra of Cur-PU-1, Cur-PU-2, and Cur-PU-4 solution (0.016mg/mL in DMSO) with in different pH.

Figure S5. The changes of mass of Cur-PU samples at different degradation time in air and upw.

Figure S6. The hemolysis test of Cur-PU-1, Cur-PU-2, and Cur-PU-4.

Figure S7. The MIC of *E.coli* and *S.aurens* of Cur-PU-1, Cur-PU-2, and Cur-PU-4 film.

Table S1. The chemical composition, molecular characteristics and TG results of Cur-PU and BDO-PU sample.

Table S2. The DSC results of Cur-PU; BDO-PU, HO-PCL-OH and HO-PEG-OH prepolymer.

Table S3. The XRD results of Cur-PU; HO-PCL-OH and HO-PEG-OH prepolymer.

Table S4. Mechanical performances, water contact angle of Cur-PU and BDO-PU samples.

Table S5. The of cyclic tensile properties of Cur-PU-3 films under different deformations and mechanical properties of the films after relaxed 24h at room temperature.

Table S6. Detailed data of the ratio ($R_f$) and recovery ratio ($R_r$) of Cur-PU and BDO-PU samples.

Table S7. The moisture content (MC) and water solubility (WS) of the Cur-PU film.

Table S8. The swelling behaviors (SR) of the Cur-PU film.

Table S9. Mechanical performances of Cur-PU samples after swelling behaviors.

Table S10. The cumulative release of Cur of Cur-PU samples.

Table S11. The UV absorption data of Cur-PU-3 solution with 150 μl of different anions. (Cur-PU-3: 0.016 mg/mL; solvent: DMSO; anions: 0.1 mol/L)

Table S12. The UV absorption data of Cur-PU-3 solution with different volumes of $OH^-$. (Cur-PU-3: 0.016 mg/mL; solvent: DMSO; $OH^-$: 0.1 mol/L).

Table S13. The UV absorption data of Cur-PU-3 solution with 150 μl $OH^-$ for different settling times at room temperature (Cur-PU-3: 0.016 mg/mL; solvent: DMSO; $OH^-$: 0.1 mol/L).

Table S14. The UV absorption data of Cur-PU-3 solution with upon the addition of different pH. (Cur-PU-3: 0.016 mg/mL; solvent: DMSO; pH: 0.1 mol/L).

Figure S15. The emission data of Cur-PU solution with in different pH. ((Cur-PU-1: 0.016 mg/mL; solvent: DMSO; pH: 0.1 mol/L).

Table S16. The changes of Mechanical performances of Cur-PU samples at different degradation time in artificial pancreatic juice.

Table S17. The antioxidant activity of Cur-PU film.

Table S18. The cell viability of C2C12 cells cultured for 24 h, 48 h, and 72 h in extracts of the of Cur-PU and BDO-PU film and the hemolysis test of Cur-PU and BDO-PU film.

Table S19. The antibacterial test of Cur-PU film.

Figure S1. The of cyclic tensile properties of Cur-PU-3 film under different deformations.

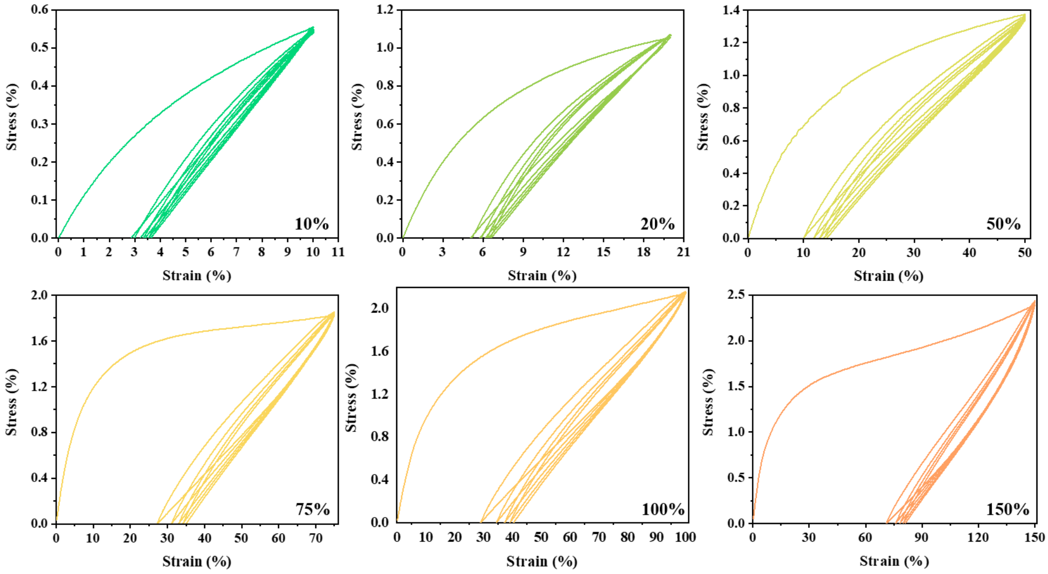


Figure S2. The moisture content (MC) and water solubility (WS) of the Cur-PU film.

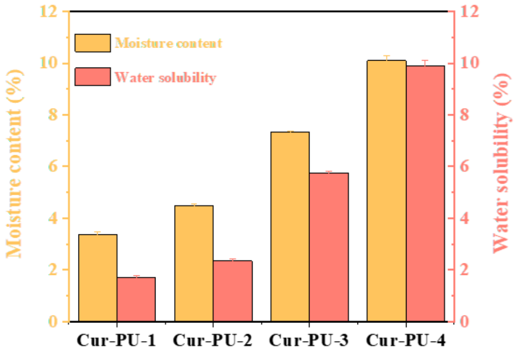


Figure S3. The UV Standard Curve of Curcumin.

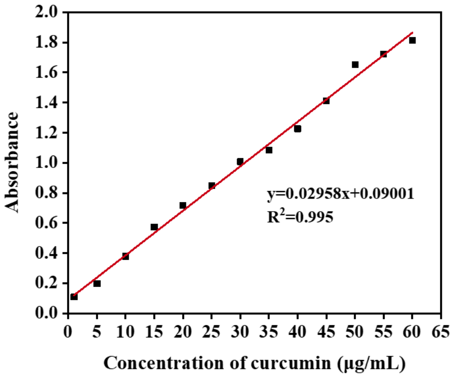


Figure S4. The emission spectra of Cur-PU-1, Cur-PU-2, and Cur-PU-4 solution (0.016mg/mL in DMSO) with in different pH.

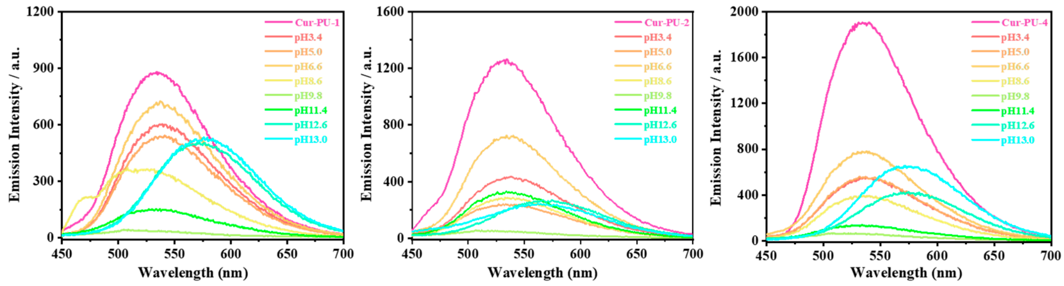


Figure S5. The changes of mass of Cur-PU samples at different degradation time in air and upw.

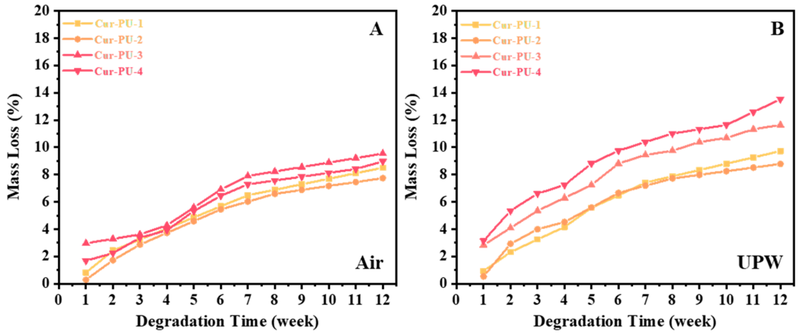


Figure S6. The hemolysis test of Cur-PU-1, Cur-PU-2, and Cur-PU-4.

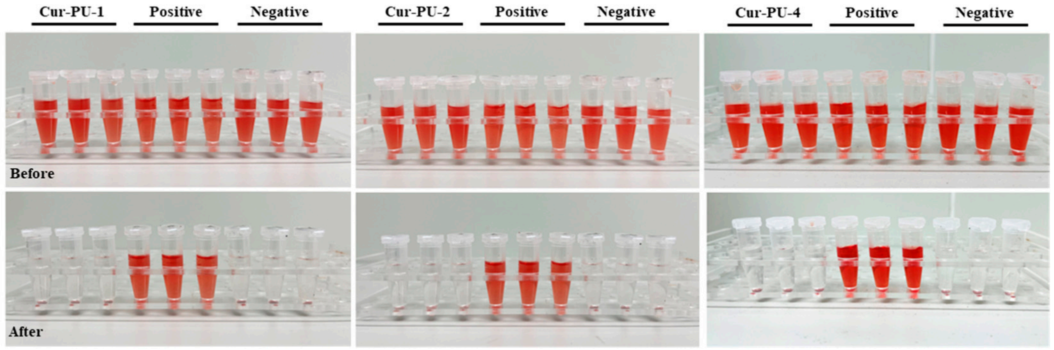


Figure S7. The MIC of *E.coli* and *S.aurens* of Cur-PU-1, Cur-PU-2, and Cur-PU-4 film.

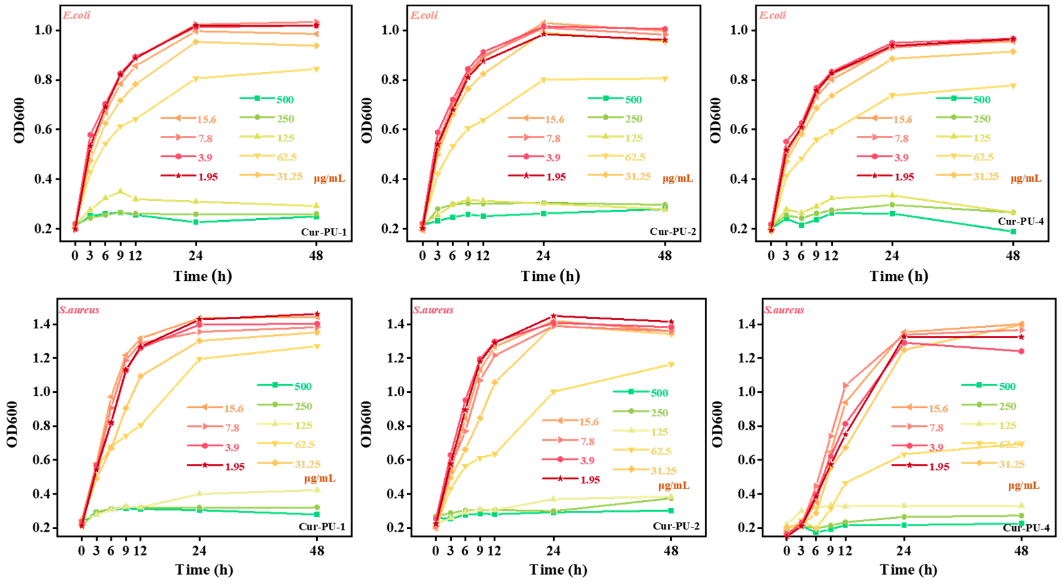

Table S1. The chemical composition, molecular characteristics and TG results of Cur-PU and BDO-PU sample.

| Sample | $n^a_{PCL}$(%) | $φ^a_{PCL}$(%) | $n^b_{PEG}$(%) | $φ^b_{PEG}$(%) | $M^c_n$(kDa) |
|---|---|---|---|---|---|
| HO-PCL-OH | 1.0 | 100 | 0 | 0 | 2.427 |
| Cur-PU-1 | 0.64 | 61.42 | 0.36 | 38.58 | 38.487 |
| Cur-PU-2 | 0.58 | 55.29 | 0.42 | 44.71 | 40.119 |
| Cur-PU-3 | 0.49 | 46.25 | 0.51 | 53.75 | 41.631 |
| Cur-PU-4 | 0.39 | 36.41 | 0.61 | 63.59 | 42.603 |
| BDO-PU | 1 | 100 | 0 | 0 | 51.713 |
| Sample | $T_{5\%}$ (℃) | $T_{50\%}$ (℃) | $T_{max}$(℃) | $M^c_w$(kDa) | Đ |
| HO-PCL-OH | / | / | / | 3.246 | 1.337 |
| Cur-PU-1 | 294.492 | 399.492 | 389.992 | 60.188 | 1.564 |
| Cur-PU-2 | 296.992 | 403.992 | 397.992 | 64.394 | 1.605 |
| Cur-PU-3 | 298.992 | 401.992 | 394.992 | 64.565 | 1.551 |
| Cur-PU-4 | 307.992 | 402.492 | 398.492 | 72.919 | 1.712 |
| BDO-PU | 288.100 | 346.830 | 356.510 | 75.332 | 1.457 |

Prepolymer: HO-PCL-OH: $M_w$ ~ 3000g/mol;

[a] $n_{PCL}$(%) and [b] $n_{PEG}$(%) measured by $^1$H NMR of Cur-PU; [a] $φ_{PCL}$(%) and [b] $φ_{PEG}$(%) calculate by (1-2):

[a] $φ_{PCL}(\%)=(n_{CL}*M_{CL})/(n_{CL}*M_{CL}+n_{EG}*M_{EG})$ (1)

[b] $φ_{PEG}(\%)=(n_{EG}*M_{EG})/(n_{CL}*M_{CL}+n_{EG}*M_{EG})$ (2)

[C]Number-average molecular weight ($M_n$) and dispersity index ($Đ=M_w/M_n$), determined by gel permeation chromatography (GPC) at 30℃ in DMF.

Table S2. The DSC results of Cur-PU; BDO-PU, HO-PCL-OH and HO-PEG-OH prepolymer.

| Sample | $T_g$ (℃) | $T_m^1$ (℃) | $H^1_m$ (J/g) | $T^2_m$ (℃) | $H^2_m$ (J/g) |
|---|---|---|---|---|---|

| | | | | | |
|---|---|---|---|---|---|
| HO-PCL-OH | -60.58 | 60.57 | 96.947 | 50.14 | 79.169 |
| HO-PEG-OH | -43.62 | 59.51 | 193.54 | 58.74 | 177.82 |
| BDO-PU | -57.01 | 51.37 | 38.905 | 48.10 | 29.542 |
| Cur-PU-1 | -54.42 | 58.36 | 47.572 | 46.04 | 39.846 |
| Cur-PU-2 | -48.69 | 51.21 | 39.838 | 44.71 | 29.954 |
| Cur-PU-3 | -49.18 | 53.73 | 49.436 | 39.72 | 51.836 |
| Cur-PU-4 | -56.57 | 42.43 | 44.871 | 37.96 | 50.210 |
| Sample | $T^1_c$ (℃) | $H^1_c$(J/g) | $\varphi^c_{PCL}$(%) | $\chi_{C,PCL}$(%) | $\chi_{C,PEG}$ (%) |
| HO-PCL-OH | 27.42 | 75.829 | 100 | 56.96 | 0.00 |
| HO-PEG-OH | 37.18 | 133.45 | 0 | 0.00 | 81.78 |
| BDO-PU | 12.22 | 34.329 | 100 | 28.25 | 0.00 |
| Cur-PU-1 | 4.33 | 35.563 | 61.42 | 46.67 | / |
| Cur-PU-2 | -9.52 | 21.236 | 55.29 | 38.98 | / |
| Cur-PU-3 | -1.92 | 20.686 | 46.25 | / | 59.14 |
| Cur-PU-4 | / | / | 36.41 | / | 48.39 |

$T_m^1$ determined from the first heating scan; $T_c^1$ determined from the first cooling scan.

$T_m^2$ and $T_c^2$ of the samples determined from the second heating scan.

The degree of crystallinity ($\chi_c$) calculated from the second heating scan.

Table S3. The XRD results of Cur-PU; HO-PCL-OH and HO-PEG-OH prepolymer.

| Sample | θ (°) |
|---|---|
| HO-PCL-OH | 21.4; 22.0; 23.7; |
| HO-PEG-OH | 19.15; 23.3; |
| Cur-PU-1 | 21.4; 23.6; |
| Cur-PU-2 | 21.3; 22.0; 23.6; |
| Cur-PU-3 | 19.05; 21.33; 22.0; 23.14; 23.6 |
| Cur-PU-4 | 19.03; 23.14; |

Table S4. Mechanical performances, water contact angle of Cur-PU and BDO-PU samples.

| Sample | Tensile strength (MPa) | Elongation at break (%) | Hardness (HA) | WCA (°) |
|---|---|---|---|---|
| Cur-PU-1 | 24.95±0.5 | 1702±23.1 | 87±3 | 91.0±0.7 |
| Cur-PU-2 | 20.84±0.7 | 1800±31.4 | 82±2 | 86.6±0.9 |
| Cur-PU-3 | 20.57±2.4 | 2222±27.2 | 78±2 | 78.1±0.9 |
| Cur-PU-4 | 15.90±1.1 | 1830±12.9 | 75±1 | 50.6±0.5 |
| BDO-PU | 39.58±0.9 | 1729±17.3 | 89±3 | 87.0±0.1 |

Table S5. The of cyclic tensile properties of Cur-PU-3 films under different deformations and mechanical properties of the films after relaxed 24h at room temperature.

| Cyclic tensile | | | Relaxed 24h | |
|---|---|---|---|---|
| $\varepsilon_m$ (%) | $\varepsilon_{ire}$ (%) | Recovery rate (%) | Stress (MPa) | Strain (%) |
| 0 | -- | -- | 20.57±2.4 | 2222±27.2 |
| 10 | 2.86 | 71.4 | 19.57±0.9 | 2578±11.1 |
| 20 | 5.09 | 74.55 | 21.71±1.2 | 2573±13.5 |
| 50 | 9.94 | 80.12 | 20.73±0.2 | 2528±22.3 |
| 75 | 27.11 | 63.85 | 21.33±0.6 | 2414±19.0 |
| 100 | 28.85 | 71.15 | 21.26±0.4 | 2533±13.3 |
| 150 | 70.86 | 52.76 | 19.71±1.3 | 2550±16.7 |
| 200 | 85.51 | 57.25 | 18.14±0.2 | 2739±10.2 |

Table S6. Detailed data of the ratio ($R_f$) and recovery ratio ($R_r$) of Cur-PU and BDO-PU samples.

| Sample | $\theta_f$ (°) | $R_f$ (%) | $\theta_r$ (°) | $R_r$ (%) |
|---|---|---|---|---|
| Cur-PU-1 | 179.9 | 99.94±0.51 | 179.2 | 99.61±0.37 |
| Cur-PU-2 | 179.8 | 99.89±1.01 | 175.0 | 97.33±0.11 |
| Cur-PU-3 | 179.8 | 99.89±0.29 | 165.0 | 91.77±0.71 |

| | | | | |
|---|---|---|---|---|
| Cur-PU-4 | 180.0 | 100.0±0.45 | 172.5 | 95.83±0.27 |
| BDO-PU | 179.82 | 99.9±0.01 | 179.64 | 99.9±0.01 |

Table S7. The moisture content (MC) and water solubility (WS) of the Cur-PU film.

| Sample | MC (%) | WS (%) |
|---|---|---|
| Cur-PU-1 | 3.36±0.11 | 1.7±0.02 |
| Cur-PU-2 | 4.49±0.09 | 2.35±0.01 |
| Cur-PU-3 | 7.34±0.07 | 5.76±0.02 |
| Cur-PU-4 | 10.11±0.03 | 9.89±0.03 |

Table S8. The swelling behaviors (SR) of the Cur-PU film.

| Time (h) | Cur-PU-1 | Cur-PU-2 | Cur-PU-3 | Cur-PU-4 |
|---|---|---|---|---|
| 0.5 | 4.00 | 13.30 | 69.50 | 114.40 |
| 1.0 | 6.00 | 20.00 | 87.69 | 150.50 |
| 1.5 | 7.99 | 26.10 | 92.78 | 166.32 |
| 2.0 | 9.34 | 28.46 | 93.62 | 171.46 |
| 3.0 | 10.45 | 30.70 | 96.46 | 174.83 |
| 4.0 | 10.70 | 31.42 | 96.73 | 175.37 |
| 5.0 | 10.61 | 31.85 | 97.06 | 176.72 |
| 7.0 | 11.07 | 31.26 | 96.96 | 174.70 |
| 9.0 | 11.23 | 31.62 | 99.39 | 176.53 |
| 12.0 | 11.52 | 32.42 | 99.73 | 177.30 |
| 24.0 | 11.11 | 32.06 | 101.18 | 177.81 |
| 36.0 | 11.84 | 33.16 | 102.33 | 180.85 |
| 48.0 | 11.84 | 32.63 | 103.37 | 183.22 |

Table S9. Mechanical performances of Cur-PU samples after swelling behaviors.

| Swelling | Sample | Stress (MPa) | Strain (%) |
|---|---|---|---|
| Original | Cur-PU-1 | 24.95±0.5 | 1702±23.1 |

| | | | |
|---|---|---|---|
| | Cur-PU-2 | 20.84±0.7 | 1800±31.4 |
| | Cur-PU-3 | 20.57±2.4 | 2222±27.2 |
| | Cur-PU-4 | 15.90±1.1 | 1830±12.9 |
| After equilibrium swelling | Cur-PU-1 | 16.62±1.3 | 1758±15.3 |
| | Cur-PU-2 | 11.90±1.7 | 1793±17.6 |
| | Cur-PU-3 | 7.67±0.9 | 2442±20.7 |
| | Cur-PU-4 | 3.96±1.4 | 2194±13.4 |
| After 100% swelling | Cur-PU-3 | 7.27±2.1 | 2441±33.9 |
| | Cur-PU-4 | 4.55±0.7 | 2559±27.1 |

Table S10. The cumulative release of Cur of Cur-PU samples.

| Time (h) | Cur-PU-1 | Cur-PU-2 | Cur-PU-3 | Cur-PU-4 |
|---|---|---|---|---|
| 0.5 | 9.260 | 11.804 | 11.442 | 19.212 |
| 1.0 | 10.291 | 13.737 | 14.044 | 22.311 |
| 1.5 | 10.703 | 13.495 | 15.183 | 23.196 |
| 2.0 | 11.941 | 15.066 | 17.378 | 25.409 |
| 3.0 | 13.315 | 17.482 | 19.899 | 26.516 |
| 4.0 | 13.728 | 17.724 | 20.794 | 25.631 |
| 5.0 | 13.865 | 17.241 | 20.631 | 28.066 |
| 7.0 | 14.003 | 18.086 | 23.396 | 30.279 |
| 9.0 | 14.209 | 19.174 | 23.884 | 30.279 |
| 12.0 | 14.278 | 20.865 | 25.185 | 29.394 |
| 24.0 | 14.347 | 20.503 | 25.754 | 30.722 |
| 36.0 | 14.003 | 20.382 | 25.429 | 30.943 |
| 48.0 | 14.347 | 19.778 | 25.971 | 30.722 |

Table S11. The UV absorption data of Cur-PU-3 solution with 150 μl of different anions.

(Cur-PU-3: 0.016 mg/mL; solvent: DMSO; anions: 0.1 mol/L)

| Anions | $\lambda^1_{abs}$ (nm) | $A^1$ | Anions | $\lambda^1_{abs}$ (nm) | $A^1$ |
|---|---|---|---|---|---|
| Original | 422.0 | 0.482 | $H_2PO_4^-$ | 435.5 | 0.484 |
| $HSO_4^-$ | 435.5 | 0.506 | $HSO_3^-$ | 435.5 | 0.483 |
| $CO_3^{2-}$ | 595.5 | 0.376 | $NO_2^-$ | 435.5 | 0.481 |
| $HPO_4^{2-}$ | 588.0 | 0.311 | $Cl^-$ | 435.5 | 0.486 |
| $SO_4^{2-}$ | 435.5 | 0.408 | $PO_4^{3-}$ | 595.5 | 0.492 |
| $OH^-$ | 498.5 | 0.743 | $HCO_3^-$ | 595.5 | 0.363 |

Table S12. The UV absorption data of Cur-PU-3 solution with different volumes of $OH^-$.

(Cur-PU-3: 0.016 mg/mL; solvent: DMSO; $OH^-$: 0.1 mol/L).

| Volumes (μL) | $\lambda^1_{abs}$ (nm) | $A^1$ | $\lambda^2_{abs}$ (nm) | $A^2$ |
|---|---|---|---|---|
| 0 | 422.0 | 0.498 | 498.0 | 0.02 |
| 10 | 422.0 | 0.326 | 498.0 | 0.042 |
| 20 | 422.0 | 0.236 | 498.0 | 0.068 |
| 30 | 422.0 | 0.182 | 498.0 | 0.100 |
| 40 | 422.0 | 0.123 | 498.0 | 0.143 |
| 50 | 422.0 | 0.112 | 498.0 | 0.207 |
| 60 | 422.0 | 0.164 | 498.0 | 0.425 |
| 70 | 422.0 | 0.250 | 498.0 | 0.632 |
| 80 | 422.0 | 0.283 | 498.0 | 0.735 |
| 90 | 422.0 | 0.267 | 498.0 | 0.680 |
| 100 | 422.0 | 0.283 | 498.0 | 0.701 |
| 110 | 422.0 | 0.288 | 498.0 | 0.719 |
| 120 | 422.0 | 0.295 | 498.0 | 0.742 |
| 130 | 422.0 | 0.295 | 498.0 | 0.739 |
| 140 | 422.0 | 0.299 | 498.0 | 0.744 |
| 150 | 422.0 | 0.297 | 498.0 | 0.730 |

Table S13. The UV absorption data of Cur-PU-3 solution with 150 μl $OH^-$ for different settling times at room temperature (Cur-PU-3: 0.016 mg/mL; solvent: DMSO; $OH^-$: 0.1 mol/L).

| Time（min） | $\lambda^1_{abs}$ (nm) | $A^1$ | $\lambda^2_{abs}$ (nm) | $A^2$ |
|---|---|---|---|---|
| 0 | 422.0 | 0.299 | 498.0 | 0.744 |
| 5 | 422.0 | 0.287 | 498.0 | 0.697 |
| 10 | 422.0 | 0.272 | 498.0 | 0.657 |
| 15 | 422.0 | 0.219 | 498.0 | 0.505 |
| 20 | 422.0 | 0.178 | 498.0 | 0.374 |
| 25 | 422.0 | 0.141 | 498.0 | 0.267 |
| 30 | 422.0 | 0.126 | 498.0 | 0.231 |

Table S14. The UV absorption data of Cur-PU-3 solution with upon the addition of different pH. (Cur-PU-3: 0.016 mg/mL; solvent: DMSO; pH: 0.1 mol/L).

| pH | $\lambda^1_{abs}$ (nm) | $A^1$ | pH | $\lambda^1_{abs}$ (nm) | $A^1$ |
|---|---|---|---|---|---|
| original | 428.0 | 0.556 | 11.0 | 428.0 | 0.463 |
| 8.0 | 428.0 | 0.547 | 12.0 | 600.0 | 0.495 |
| 9.0 | 428.0 | 0.552 | 13.0 | 498.0 | 0.73 |
| 10.0 | 428.0 | 0.555 | 14.0 | 498.0 | 0.784 |

Figure S15. The emission data of Cur-PU solution with in different pH. (Cur-PU-1: 0.016 mg/mL; solvent: DMSO; pH: 0.1 mol/L).

| pH | Cur-PU-1 | | Cur-PU-2 | | Cur-PU-3 | | Cur-PU-4 | |
|---|---|---|---|---|---|---|---|---|
| | $\lambda_{em}$ (nm) | FL | $\lambda_{em}$ (nm) | FL | $\lambda_{em}$ (nm) | FL | $\lambda_{em}$ (nm) | FL |
| original | 534.0 | 881.2 | 534.0 | 1266 | 534.0 | 1367 | 534.0 | 1906 |
| 3.4 | 539.0 | 605.3 | 538.0 | 435.6 | 539.0 | 997 | 538.0 | 553.2 |
| 5.0 | 541.0 | 542.7 | 541.0 | 240.3 | 541.0 | 746.3 | 532.0 | 556.5 |
| 6.6 | 537.0 | 724.2 | 534.0 | 728.9 | 539.0 | 1085 | 538.0 | 780.1 |

| | | | | | | | | |
|---|---|---|---|---|---|---|---|---|
| 8.6 | 526.0 | 365.8 | 529.0 | 284.7 | 538.0 | 495.3 | 538.0 | 394.5 |
| 9.8 | 525.0 | 40.66 | 524.0 | 53.61 | 529.0 | 86.58 | 528.0 | 63.98 |
| 11.4 | 527.0 | 152.4 | 526.0 | 326.9 | 532.0 | 505.4 | 534.0 | 137.9 |
| 12.6 | 562.0 | 500.3 | 564.0 | 264.1 | 562.0 | 621.4 | 568.0 | 422.1 |
| 13.0 | 560.0 | 498.6 | 563.0 | 246.6 | 566.0 | 659.1 | 571.0 | 654.9 |

Table S16. The changes of Mechanical performances of Cur-PU samples at different degradation time in artificial pancreatic juice.

| Time (week) | Cur-PU-1 | | Cur-PU-2 | |
|---|---|---|---|---|
| | Stress (MPa) | Strain (%) | Stress (MPa) | Strain (%) |
| 0 | 24.95±0.5 | 1702±23.1 | 20.84±0.7 | 1800±31.4 |
| 1 | 12.933±0.3 | 1246±20.9 | 13.26±0.3 | 1532±12.6 |
| 2 | 14.915±0.2 | 1281±13.7 | 8.835±0.4 | 1338±13.2 |
| 3 | 13.396±0.3 | 1328±18.5 | 8.811±0.3 | 1259±21.4 |
| 4 | 13.021±0.1 | 1196±10.2 | 7.754±0.1 | 1221±23.6 |
| 5 | 12.283±0.4 | 942±9.3 | 7.678±0.4 | 1212±19.6 |
| 6 | 11.596±0.8 | 1056±12.1 | 7.134±0.6 | 1249±10.2 |
| 7 | 11.264±1.1 | 1080±10.9 | 7.064±0.2 | 1232±9.1 |
| 8 | 11.265±1.3 | 877±11.3 | 6.388±0.5 | 991±8.7 |
| 9 | 9.685±0.9 | 787±12.4 | 5.495±0.1 | 953±5.5 |
| Time (week) | Cur-PU-3 | | Cur-PU-4 | |
| | Stress (MPa) | Strain (%) | Stress (MPa) | Strain (%) |
| 0 | 20.57±2.4 | 2222±27.2 | 15.90±1.1 | 1830±12.9 |
| 1 | 16.569±1.9 | 2825±22.8 | 12.594±1.4 | 1896±21.2 |
| 2 | 11.738±2.3 | 2336±31.1 | 6.141±0.7 | 1547±10.7 |
| 3 | 10.126±3.1 | 2164±13.4 | 5.923±1.2 | 924±16.3 |
| 4 | 10.255±0.9 | 2101±18.2 | 3.765±0.6 | 798±11.1 |

| | | | |
|---|---|---|---|
| 5 | 9.786±0.7 | 1212±22.9 | The Cur-PU-4 film has fractured and is thus unavailable for mechanical property testing. |
| 6 | 9.588±1.3 | 1249±17.3 | |
| 7 | 9.444±2.1 | 1232±12.6 | |
| 8 | 8.122±0.8 | 991±13.3 | |
| 9 | 7.979±0.3 | 953±11.7 | |

Table S17. The antioxidant activity of Cur-PU film.

| Sample | DPPH scavenging activity (%) | | |
|---|---|---|---|
| | 1h | 24h | 48h |
| Cur-PU-1 | 0.44±0.091 | 0.87±0.101 | 1.22±0.123 |
| Cur-PU-2 | 1.93±0.012 | 4.25±0.213 | 6.20±0.110 |
| Cur-PU-3 | 3.96±0.071 | 10.50±0.192 | 20.50±0.220 |
| Cur-PU-4 | 7.95±0.029 | 17.11±0.095 | 47.78±0.400 |

Table S18. The cell viability of C2C12 cells cultured for 24 h, 48 h, and 72 h in extracts of the of Cur-PU and BDO-PU film and the hemolysis test of Cur-PU and BDO-PU film.

| Sample | Cell culture time (h) | | | Relative hemolysis rate (%) |
|---|---|---|---|---|
| | 24h | 48h | 72h | |
| | OD (%) | OD (%) | OD (%) | |
| Cur-PU-1 | 94.97±1.92 | 99.01±1.03 | 98.78±1.34 | 0.94±0.00113 |
| Cur-PU-2 | 99.02±0.83 | 97.71±0.89 | 98.82±2.01 | 1.02±0.00809 |
| Cur-PU-3 | 98.23±1.02 | 99.29±1.19 | 95.49±1.73 | 1.02±0.01002 |
| Cur-PU-4 | 96.58±1.27 | 99.79±1.21 | 96.94±0.82 | 0.59±0.01024 |
| BDO-PU | 96.27%±1.02 | 93.75%±0.98 | 100.84%±2.09 | 1.68±0.077 |

Table S19. The antibacterial test of Cur-PU film.

| Sample | Antibacterial circle diameter (mm) | |
|---|---|---|
| | *S. aureus* | *E. coli* |
| Control blank | 7.90±0.038 | 8.31±0.068 |

| | | |
|---|---|---|
| DMSO | 12.20±0.208 | 10.01±0.260 |
| Cur-PU-1 | 12.54±0.415 | 9.94±0.451 |
| Cur-PU-2 | 13.16±0.429 | 10.86±0.275 |
| Cur-PU-3 | 14.77±0.663 | 11.28±0.219 |
| Cur-PU-4 | 16.21±0.645 | 11.72±0.179 |
